# Materia Medica—A Science, Its Future, Uses, and How It Is to Be Used

**Published:** 1884-09

**Authors:** P. P. Wells


### HOMŒOPATHIC PHYSICIAN.

A MONTHLY JOURNAL OF MEDICAL SCIENCE.

"If our school ever gives up the strict inductive method of Hahnemann, we are lost, and deserve only to be mentioned as a caricature in the history of medicine."—CONSTANTINE HERING.

Vol. IV. SEPTEMBER, 1884. No. 9.

### MATERIA MEDICA—A SCIENCE, ITS NATURE, USES, AND HOW IT IS TO BE USED.

Materia medica is one of the sciences supposed to belong especially to the domain of practical medicine, and the knowledge of it to be of chief interest to those who have given their lives to the work of healing the sick. It embraces all the knowledge we have of the action of certain agents on the organism of man, changing the functions of its organs so that these, while under the power of these agents, are found in departure from that harmony of action we call health, the agents being thus declared to be sick-making substances. A knowledge of these substances, as belonging to the domain of materia medica, whenever obtained, discloses the fact that each agent disturbs this harmony in a manner peculiar to itself, in this differing from the action of all other sick-making powers. There may be found in the effects of any one of these many similarities to effects of other agents, but there will be differences in each which are found in no other, and these are the facts of greatest importance in all clinical use of knowledge of materia medica.

A knowledge of the commercial and natural history of these agents brings them into relationship to other natural sciences—a knowledge of their sick-making powers into relation to the science of therapeutics, and it is this relation with which we, as practicing physicians, are chiefly concerned. *In what manner* does each agent affect each function and tissue? This is the first inquiry concerning it. The discovery of this fact, or of these facts and their records, and we have our armamentarium ready for practical duties.

In bringing any member of the family of our materia medica to the relief of the sick, before its use it should always be remembered we are about to introduce into the clinical problem before us a new and additional sick-making force—and further, if the agent selected be that required by our law, it not only makes sick, but makes a sickness *like* to that we are now about to attempt to cure. If this be borne in mind, it will be apparent to the dullest apprehension that any excess of this agent over so much of it as the cure requires must go to the increase of the sick condition we are to cure. Hence, it can never be a question of small importance, when we have found the true specific for our case, as the law requires, which we decide when we answer—*how much* of it? The answer is ever as stated by our excellent friend, J. W. Dowling, M. D., in his reply to Dr. Palmer—"The least possible quantity which will cure." The cure being our only possible objective in the use of the agent, all in excess of that needed for gaining this end must be superfluous at least, and must always be attended by risk of great and positive injury.

The science of materia medica in its practical relation to therapeutics is presented to us in two very different aspects at the present day, according as it is regarded from the standpoints of the allopathic or homœopathic schools. The former regards the agents it employs in the material mass, and answers the question, How much? by another, How much will he bear?—*i. e.*, How much can he take and escape with life? He guesses at this, and then it too often happens the patient does not escape—the doctor guessed wrong. On the other hand, the homœopathist deals, not with the material mass of the agent, but with its developed and liberated spirit, and answers the question, after selecting his curative, How much? by this other—How little and in what form will it best accomplish the cure? The question, How much? brings neither to his mind nor to his case any shadow of suspicion of risk, and we may add, there should be just as little of *guessing*—which makes so great a figure in old school therapeutics. In selecting his remedy or remedies, the old school prescriber *guesses*. The homœopathist, on the other hand, before the use of any drug, should be in a position to say, as to its selection and dose—I *know*.

Then, as before the two schools, materia medica as a science presents very different aspects. The old school recognizes the origin, physical properties of the drug, and its more particular modification of some *one* function of some particular organ, and so he calls the substance a cathartic, an emetic, etc., according as it may have shown preference for one organ or function rather than for others. And on this foundation he makes the science one of classification, and from this deduces its nomenclature. On the other hand, the homœopathic school receives the record of *all* the ascertained effects on the human organism of all the drugs embraced in its materia medica, in their multitudinous details, on each and every function, bodily and mental, and accepts these in their entirety as the expression of the sum of its science of materia medica. So it is found constituted of *known facts,* neither theory nor guessing making the slightest part of it. It stands, thus, a whole, without the classification or nomenclature which characterizes that of old school medicine.

It is equally noteworthy that in the practical use of this science the methods of the two schools are equally diverse. The old school prescriber has reference to one property of his drug, and relies for all beyond this of change for good to his patient on an expected indirect result of its use, as in revulsions, diversions, counter-irritations, etc. The homœopathist, on the other hand, takes into view in his prescribing every part of the sick phenomena of his patient and all those of the ascertained effects of the drug, and so selects the one which in these presents the greatest likeness to those of the other, and so, by direct and specific impress on the affected organs and functions, he secures restoration to normal action and conditions without any of the revolutionary experiences which constantly characterize the practical attempts at healing by the therapeutic means and methods of the old school. In this contrast is seen the radical difference between the philosophy and science of the two schools—the philosophy of the therapeutics of the old school so largely made up of theory and guessing, that of the new consisting of an established law, and while the application of the therapeutics of the old is made up so almost exclusively of indirect methods and means, that of the new is wholly specific and direct.

But now to deal more particularly with the science of materia medica as characteristic of our own school: We find lying near the beginning of endeavor to a thorough understanding of it the question, What is it in the drug agent which makes men sick? This power which cures—what is it? A right answer to this and a right understanding of its nature may be set down as indispensable to best practical results from its clinical use. We have said Homœopathy "deals not with the material mass of the drug, but with its developed and liberated spirit." By this we mean it deals with that something in the drug which has power to make human organs and functions sick. If there be an objection to the use of the word *spirit*, we will not make a quarrel with any man for the word who will bring us one which better expresses the nature of that power which has been planted in and associated with the drug, and which we have so abundant evidence is something distinct from the material substance of the drug.

It is a something which admits of separation from the matter of its original association, and of being transferred to other forms of neutral matter, retaining in this new association all its original sick-making power, and by artificial manipulation of having its curing power immensely enhanced, and this to a degree not at all to be accounted for by any supposed divisibility of the matter of the drug, or by any actual division of its constituent molecules. This something, whatever it is, we have called spirit because we had no better word. We mean simply to characterize this something as wholly distinct from all forms of matter. It will be seen that each example of this power has stamped on it its own individuality of character, and it is not a little interesting to see how this individuality is retained when the power has been transferred to other and neutral forms of matter.

In speaking of this power we have used the word *quantity* in deference to the general impression that this belongs to all ideas of medicinal doses in practical therapeutics. Practically this is wrong. For doses of medicinal agents do cure sick persons in a form where all idea of *quantity*, as referring to any sum of the originally associated drug matter, is in the highest degree absurd, and sometimes they cure surprisingly well. They cure often, in the hands of those who know how to use them, sicknesses which have failed to respond to doses of the same drug in forms where the idea of minutely divided molecules of the drug might have been conceived of as a physical possibility, thus demonstrating the immaterial nature of the curing force, and the enhanced power of that force by the process of expansion. Facts, in more than necessary abundance, seem to demonstrate, in this series of cures, the truth of increase, development, and liberation of a spirit power wholly unmistakable, even by the least experienced practical observer. We do not forget these cures have been scouted at by those who have never seen them, and out and out denied to have had any existence save in the imagination of fanciful men, but before the *fact* of such cures, which we have seen many times, we can perceive no good reason for respecting the folly of the scoffer or the ignorance of their negators. It should not be forgotten by any one who seeks to add to his knowledge of yesterday by observation of new facts to-day, that neither scoffs, incredulity, nor denial have the least power to destroy facts or annihilate their witness to truths to which they are related. These may prevent the weak or the prejudiced from accepting this testimony, but then what is the result, and whose is the loss? The weak are only made weaker by rejecting the truth, while the prejudiced are only consigned to a more complete blindness.

We regard a right understanding of the nature of sicknesses and of that of their God-given curing agents as lying at the very foundation of the best possible practical successes of healing. To continue to regard sicknesses as *things*—and things somewhat distinct and separate from the sick individual—is to stultify one's self in the murk and ignorance of remote antiquity; and to build up notions of these things into forms, classes, genera, and species, like material objects in natural history, calling them *diseases*, with fanciful names, and dwelling on these and imagining them as possessed, each, of a nature and form quite its own, and calling this by the well-sounding word, so charming to many, *pathology*, is the weakness and the folly of dominant medicine to-day. To affect a following of these false and foolish notions, as some of our school seem given to do, leaving for this the truth given us by our truly great master in all that constitutes true philosophy of disease and cure, can only be paralleled by the man who forsakes the clear light of noon for the murk of midnight and affirms he finds this clearer, brighter, and more pleasant, safe, and useful for all the practical purposes and duties of this life. This is the kind of pathology which old school physic has declared, from the beginning till now, that Hahnemann had none of it. And what then? It has only been the better for him and for Homœopathy that he had none. He had a better pathology, which old physic has refused to consider or accept, and which is all too little understood by many of his professed followers. Fashion, prejudice, and the force of example have been only too powerful in turning away practical healers from the simple truth, plain, clear, bright, and beautiful, as he has given it to us in his philosophy of diseases and their curatives, which he called *Homœopathy*. He taught that sicknesses were only modified life force and consequent modified function, and not *things* at all. That the sum of these modifications, which we are accustomed to call "the totality of the *symptoms*," is the whole of *pathology* of any sickness which any man can or need know. And we may be perfectly assured that any so-called pathology, outside of, or in addition to this, has its existence mainly in the imaginations of men less wise than they think themselves to be, and is, except as curiosities of human invention, worthy of little consideration and less respect.

Then, as to the nature of the curing force. This is no more a material thing than is the sickness it cures. It is a force and nothing more. To conceive of it, speak of it, or employ it as matter is only a departure from the proprieties which belong to truth, the tendency of which is to injure the chances of recovery, and perhaps, to effectually exclude from the case under treatment the very principles on which best possible success is founded.

If it be asked, How do we know this which cures is not material? we answer, We know this because it does not obey the laws which govern matter, one of which it violates in every day's experience. It is this, when force is derived from matter, the sum of the force is determined by the sum of the matter, *i. e.*, the greater the sum of the matter the greater the force. Now, there are constantly occurring cures where, if matter be predicated of the curing doses, there must have been most of the matter where the least of curing power was experienced, or even none at all, but doses in which there must have been much less of matter have effected prompt, perfect, and permanent cures, so, if the curative were matter, reversing the law which determines the sum of the force by the sum of the matter. If this which has and does so cure be matter, we may truly say there is in the material world no other similar violation of this law.

Then this curing force is found acting as no form of matter does in this: Place a single medicated pellet or any small number of such in a phial, and fill it with unmedicated pellets, and these will all soon become medicated with the same healing force as was in the original medicated one, while at the same time they have received no new property of matter nor any addition to those they possessed when they were but blanks. Now we know of no instance, nor can we conceive of any, where matter in form passes from one material body to another, imparting to this new properties, and neither of them experiencing in the process any change of loss or gain in properties or qualities belonging to them as matter. This certainly has been many times observed as to this curing force, and each time it has given its own nature to the new pellets it has clearly demonstrated that nature to be non-material.

Then, if the curing power be but force and not matter, we may safely dismiss from our thoughts and practice all ideas of *quantity* as pertaining to the dose given for any cure, and especially that false and foolish notion—so hard to disabuse the inexperienced mind of—that a greater dose of a curative must impart greater curing power to the patient. It is the specific nature and relationship of the dose which cures, and not the quantity given. The cure is like the cause; it cures because it is its nature to cure this particular case, and not because of any amount of it that has been given. The cause—be it smallpox—increasing the sum of the poison, only produces smallpox, and never yellow fever or any other form of sickness; and curious enough and worth remembering it is, that increase of the quantity of the poison does not intensify the sickness it has caused, but just the reverse, as is shown in the history of the practice of inoculation, which was a successful resort—or was supposed to be—to secure a mild example of the disease as compared with that contracted from the poison diffused in the atmosphere, the less quantity of the poison producing the intenser morbid action.

Now, having looked at materia medica as a science—to its nature and uses, and also to the nature of the objectives of these uses—we come to the question: *How* shall the materia medica of our school be used so that the greatest practical successes may be attained by its practical healers? The question is met at the outset by differences of judgment and practice and by no small measure of excited feeling at times, even to an extent which makes its dispassionate discussion impossible. This we judge to be both unreasonable and wrong. It ought always to be permissible to elicit truth. Excited feeling, especially if this be inspired by prejudice in any degree, is not a profitable element to bring into this work. And as, in answering this question—of the greatest possible importance to doctors and men—the simple truth alone is all which can be of the least value to either, let us look at it in the clearest light we can bring to bear on it.

In the first place, then, let us keep in mind the nature of sicknesses, not *things*, not material, but only a modified action of force, and also the nature of the curing agent—itself not material, but a liberated and developed force—as given to the homœopathic healer, who now has before him an example of this sick acting force he is expected to cure. What shall he do? and how shall he do it? Homœopathy cures mainly by the use of drug forces. In this there is unanimity with its practitioners. But in what form shall these forces be employed in the case before us? In this decision unanimity is wanting. The facts of the case being the same for all, why should there be diversity of judgment among healers as to the methods and means to be employed for gaining most beneficent results most speedily and most safely? Equal intelligences will be very likely to select the same agent for the cure, but these may differ greatly as to how the agent shall be used. Why is this? and is it necessary? A. believes the best cure will be made by giving the crude drug or preparations but little removed from this. He is accustomed to give these because he believes them best, and therefore he has no experience of other methods and has no confidence in them —especially he has none in liberation and development of force— and there is no reason why he should have. He knows nothing of them. Believing, in addition, that he knows as much as his neighbors, he tolerates no difference of opinion and will listen to no discussion. He believes the curing power to be a material thing, and so his judgment has failed to be enlightened by acceptance of true homœopathic philosophy of the curing power. It may also happen that he takes the material view of diseases, and talks pathology, and of these as things distinct from the suffering patient, and regards himself as a votary of "scientific medicine." These may be reasons why he attempts cures with crude drugs. They are sufficient reasons, if present, for regarding him as wholly unenlightened by true homœopathic philosophy.

B. does not agree with A. in his views of drug power. He partially accepts the idea of development—that is, development to some extent, but limits this in the drugs he uses to a comparatively low range of numbers, and beyond these he has no experience and therefore no confidence. He has never parted from the idea that it is drug matter with which he is dealing, and apart from this he has no conception of curing power. He may have less of an idea that he is to treat a *thing*, which he calls a disease, and talk less of its *pathology* than A. but as compared with him he has only partially emerged from the errors and darkness which will always, wherever met, be found an impediment to best successes in healing. He must see it possible that there is *some* matter of the drug in his doses. He is a votary of divisibility. He in a measure holds with one hand somewhat to old school notions, while with the other he feels after principles of the new, hoping to find some good in them. He believes there is good in them, but he finds it difficult to get at it, and this because his attention is greatly occupied by the idea that there must be something for him to retain of old school physic, and this it may be it is which blinds his eyes. Certain it is, that the more he retains of this the less he will see of the beauty and value of the new.

C. differs from both. It may be he has passed over the ground of both A. and B. and seen just where each is in error as to all pertaining to true homœopathic philosophy and practice founded on it. He began, it may be, with the law of similars —he would try and test this. Then, if he found evidence in such trials that this was indeed a principle in the nature of things, and if, perchance, he had read the *Organon* before he began these trials, he may have asked himself, May there not be something in liberation and development of drug force too? He determined to try this for himself, but he would do this very carefully. He would not allow himself to be fooled by any man's fanciful notions, so he began with doses where this development was but small. Perhaps the third number was sufficient for his beginning. This he found active, and may have thought, perhaps even higher numbers may prove active also. He tried them and found them not only active but curative in greater degree than those lower ones he had previously used, which most assuredly he would, if he followed the directions of the *Organon*, making the totality of the symptoms the basis of his prescription. If, instead of this, he prescribed for names or imagined internal conditions, for pathology's sake, he just as certainly learned nothing at all. If he continued to prescribe on symptoms, he found the higher the numbers he used, the required likeness between the sickness and the drug being present, the more speedily and perfectly they cure, and so through this path of observation and experience he has come at last to the use of what have of late come to be spoken of as "*high potencies.*" He has come to the use of the immaterial, liberated, curing power for relief of immaterial sick functions, and has realized successes such as he never had seen before. He has found the related curing forces not only like the sick-making forces in their actions on the organism, but like also in their nature in this, that both are immaterial. He may have found it difficult to divest his mind of impressions which were educated into him as to the nature of sicknesses and their curatives, diseases having been treated of, and treated as invaders of the organism, dwelling in it, with pain and destruction as their consequences, something in the organism but not of it, and that the more violent the action they here set up, the fiercer and more ponderous were to be the blows required for their expulsion. It was but natural that this view of sicknesses should have been accompanied by a view of the nature of curing agents correspondent to it; that to cure violent diseases ponderous doses of mighty drugs were alone equal to the task and only to be depended on. It is not an easy work, nor the work of a day, getting rid of such impressions, false as they are. Have they not come from teachers and books who have had our reverence? Are they not the only remaining fruits of that reverence? Have these not told us, and told us at all times, that the true answer to the question as to the dose, How much? is found in the other, How much will he bear? And did they not know, these who have been since the days of our pupilage our ultimate authorities? Have we not been virtually taught that the patient was to be permitted to escape with his life, and that this was all he had a right to expect at the hands of his prescriber, who began his clinical duties with the inquiry, "How much will he bear?" Verily, sometimes the poor victims bore a good deal,* and then sometimes did escape! It is not a work of ease, or the work of a day, to escape from prejudices and teachings like these. And then, it may be, the poor man who is struggling for this freedom has to meet another and powerful influence impelling him to remain in the philosophy and practice of his teachers. He meets ridicule, perhaps of himself personally, certainly of the results and convictions he has realized from his experiments, and ridicule is often harder to face than arguments. His experiments have given him facts. These cannot be gainsaid or set aside by other facts, but they can be met by this devil's argument, ridicule, and as his opponents have this and nothing more, they resort to it as best they may. Ridicule, especially from good men, is hard to bear, and even good men in all else have not scorned resort to this in attacks on God's law and its logical use, so different from all pertaining to their own teachings and practice.

---

*The following prescription was read on the files of a retail apothecary, in a village near Boston, by the writer in his early professional life. It was written by one who stood high in that city with those who stood highest in public estimation as healers:

℞. Ol. Ricini ℥ ii

Bals. copaibæ, ℥ i

Kreosoton, ʒ ii

Mix; dose a teaspoonful.

Who was to take this? I asked of the apothecary. J. A. B's baby, was the answer. The baby was two weeks old; was a grandniece of our late excellent Governor Dix, and did not die! I repeated the prescription and the facts to my most excellent professor of thoery and practice, telling him the little patient survived the dose. In his own peculiar quiet way he remarked: "*I have always regarded Dr. S. as a remarkably fortunate practitioner!*" This time the Doctor had a very fortunate patient.

In all this the man who will so use this law and the appliances it requires for the cure of the sick has more than an equivalent for all his struggles and all possible opposition, in his practical successes, and the approval of a good conscience, and any one may share these with him who will do, as he presumably has done, in coming to this right use of our materia medica. He has only to follow Hahnemann's direction:

"*Mach's nach, aber, mach's genau nach.*"

Do it *exactly* as I have done. P. P. WELLS.

